# An analysis of controlled human infection studies registered on ClinicalTrials.gov

**DOI:** 10.1136/bmjopen-2024-085250

**Published:** 2025-02-07

**Authors:** Danny Toomey, Jupiter Adams-Phipps, James Wilkinson, John Pietro, Jake Littman, Steffen Kamenicek, Daniel Kaufman, Joshua Osowicki, Meta Roestenberg, Ian J Saldanha, Euzebiusz Jamrozik

**Affiliations:** 1 1Day Sooner Research Team, Claymont, Delaware, USA; 2 Geisinger Commonwealth School of Medicine, Scranton, Pennsylvania, USA; 3 University of Pittsburgh School of Medicine, Pittsburgh, Pennsylvania, USA; 4 Tropical Diseases Research Group, Murdoch Children's Research Institute, Parkville, Victoria, Australia; 5 Leiden University Medical Center, Center for Infectious Diseases, Leiden, Zuid-Holland, Netherlands; 6 Department of Epidemiology, Johns Hopkins University Bloomberg School of Public Health, Baltimore, Maryland, USA; 7 Ethox and Pandemic Sciences Institute, University of Oxford, Oxford, UK; 8 Department of Infectious Diseases and Royal Melbourne Hospital Department of Medicine, University of Melbourne, Melbourne, Victoria, Australia

**Keywords:** STATISTICS and RESEARCH METHODS, MEDICAL ETHICS, Clinical Trial, Adverse events, Public health

## Abstract

**Abstract:**

**Objectives:**

Controlled human infection studies (CHIS) involve intentional exposure of human volunteers to infectious agents. A previous systematic review found that adverse event (AE) reporting across CHIS is inconsistent, which makes data aggregation and reuse difficult. We hypothesised that data may be more easily aggregated using a clinical trial registry such as ClinicalTrals.gov, the largest publicly accessible registry of clinical trial data. The objectives of the current analysis were to (1) evaluate the use of ClinicalTrials.gov for CHIS data reporting and (2) compare CHIS clinical trial participant flow and AE reporting in ClinicalTrials.gov with the same data in corresponding published articles.

**Design:**

ClinicalTrials.gov records that described a CHIS were included and data were accessed using the Aggregated Analysis of ClinicalTrials.gov application programming interface. These data were compared with results extracted from publications associated with included records’ NCT identifiers and compared in groups stratified by sponsor type, cohort size and risk of bias. Results were considered discrepant if the same number was reported unambiguously differently in the clinical trial record and its associated publications. The frequencies of these discrepancies were used to quantify the differences between reporting in ClinicalTrials.gov records and publications of the same results.

**Results:**

We screened 5131 ClinicalTrials.gov records for inclusion, reviewed 410 records in full and included 344 records. The overall prevalence of any discrepancy was 40%. Compared with their respective groups, significant discrepancies were observed in publicly funded trials, trials in the third quartile of study size and trials with a high risk of bias in selection of the reported result. Five studies reported a total of five serious AEs in ClinicalTrials.gov records but not in any associated publications.

**Conclusion:**

We identified an overall prevalence of discrepancy of 40% in CHIS, which is comparable with the prevalence observed in other types of clinical trials. In general, medium-sized, publicly funded trials tended to have more discrepancies in reporting, which may reflect the resources typically available to large, privately funded trials or the relative ease of reporting in smaller trials with fewer overall AEs. These results highlight the need to facilitate clear and consistent reporting in CHIS.

**PROSPERO registration number:**

CRD42022330047.

**STRENGTH AND LIMITATIONS OF THIS STUDY**

- This is the first study comparing controlled human infection studies adverse events reporting with trial registry data.
- This study contributes to a sparse general literature on reporting discrepancies.
- We provide recommendations for best practices to reduce the problems we identify.
- Our data likely exhibit heterogeneity arising from aggregation across studies of different infectious agents.
- Only ClinicalTrials.gov was evaluated, and it is possible that trials that would have met inclusion criteria are registered in other databases.

## Introduction

 Controlled human infection studies (CHIS) model an encounter between human hosts and pathogens by deliberately exposing selected volunteers to a well-characterised infectious agent under controlled conditions.^1 2^ CHIS are used for many purposes, such as studying the transmission and pathogenesis of infectious diseases and evaluating the efficacy of vaccines or other interventions.^3^ Records of such trials date back to the 18th century, although many early challenge experiments would not have met modern ethical research standards set forth in the 1970s.^4^ Although CHIS are a powerful tool that can be used to expedite the development of vaccines and therapies for infectious diseases,^15^,^7^ their use has been relatively sporadic, potentially reflecting ethical or generalizability concerns or a lack of sustained investment.^3 4 8^ The benefits of the data gathered in CHIS would be enhanced by comprehensive reporting as well as appropriate standardisation of study protocols. Additionally, the use of data-sharing principles, such as Findability, Accessibility, Interoperability and Reuse (FAIR), greatly aids in the aggregation and reuse of CHIS data, which is vital due to their smaller size compared with other clinical trials. The average size of CHIS is 55 volunteers, which is lower than the average size of 1262 volunteers for interventional trials listed in ClinicalTrials.gov.^9^,^11^

To investigate the safety of modern CHIS, we previously conducted a systematic review of adverse events (AEs) and serious AEs (SAEs) in peer-reviewed publications describing CHIS between 1980 and 2021.^10^ SAEs are defined by the US Food and Drug Administration (FDA) as an AE that results in serious outcomes, such as hospitalisation, permanent disability and death.^12^ Some AEs that occur during a study may be directly related to study interventions while others may be incidental. Our previous review found that a minority of participants in modern CHIS experienced challenge-related severe AEs (as defined by study authors) or SAEs.^10^ Across 187 studies that reported SAE data, 23 of 10 016 participants (0.2%) experienced at least one SAE. The most frequent SAEs were severe vomiting and/or diarrhoea, hepatitis and hyperbilirubinemia. Across 94 studies that reported data on severe AEs (grade 3 or higher), between 285 and 801 out of 5083 participants (5.6%–15.8%; A range of values was given to account for unclear data reporting in some studies) experienced at least one severe AE.^10^

Although the above findings generally support the safety of modern CHIS, the review also identified issues related to nonstandardised reporting of CHIS,^10 13^ including those regarding the classification of this type of research. Previous work has discussed the wide-ranging terminology in use for CHIS,^13^ with the predominant issues posed being difficulty identifying CHIS across different models and fields. This ambiguity is related to a lack of consistency with what constitutes a ‘challenge’ with a microorganism. Some intuitive definitions such as defining a challenge study as involving deliberate infection with a known infectious organism might be perceived to exclude studies studies using live-attenuated vaccines as a challenge agent and might not exclude studies of infection as therapy. Studies aiming to produce colonisation rather than symptomatic infection might be considered another borderline case. The review also found inconsistent use of trial registries,^10^ with approximately 75% of CHIS started in the 2010s listed in at least one registry.

ClinicalTrials.gov, the world’s largest repository for clinical trial data, was launched in February 2000 by the US National Institutes of Health (NIH) and the FDA as a public registry of medical studies in human volunteers, and is maintained by the US National Library of Medicine.^14^,^16^ Database fields detailing study AEs were made publicly available in September 2008.^14^ Although the rate of study registration has increased over time as a result of regulatory requirements and voluntary registration on the part of sponsors and investigators,^14^ some studies still do not get registered on ClinicalTrials.gov or any other registry. Despite requirements for NIH-funded trials to post results within 1 year of study completion, many fail to do so, with a recent report identifying over 3000 clinical trials across all fields with missing results that have been overdue since February 2018.^17^

A key finding of our previous review was that AE reporting is often unclear or missing in CHIS publications.^10^ The consequences of this are twofold: the experiences of volunteers are omitted from the research record, which arguably violates the standard of Respect for Persons set by the Belmont Report, and the data that these volunteers contribute are unable to be reused by other researchers, minimising their contribution to science. To understand whether AE reporting is more clear or complete in ClinicalTrials.gov records than in publications, the current analysis (1) evaluates the use of ClinicalTrials.gov for CHIS registration and data reporting and (2) compares CHIS clinical trial participant flow and AE reporting in ClinicalTrials.gov with the same data in corresponding published articles. Our rationale for these objectives was to be able to quantify the frequency of discrepancies at each level of an CHIS participant flow, which we had previously observed but lacked concrete data for analysis.

## Methods

A full protocol is available in the online supplementary materials. Patients and members of the public were not involved in the design of the study. This analysis was preregistered at PROSPERO (CRD42022330047).

### Eligibility criteria for CHIS

Studies registered on ClinicalTrials.gov that involved intentional exposure of human volunteers to an infectious agent to develop or use a model of infection, commonly known as CHIS or human challenge trials, were included. There is ongoing debate regarding the precise definition of an CHIS.^13 18^ For our review, we examined studies that involved intentional exposure of human volunteers to wild-type or attenuated organisms (infectious agents). Studies in which previously challenged participants were challenged again with the same infectious agent (ie, rechallenges) were included. Studies involving live-attenuated vaccines were only included when the vaccine strain was used as a challenge agent. Records that posted results and included at least one associated publication were included in the analysis.

### Identification of CHIS registered on ClinicalTrials.gov

Searches were performed as structured language queries using the Aggregated Analysis of ClinicalTrials.gov application programming interface (AACT API) on 15 April 2023. The search included terms for challenge, infection and controlled experimental studies, filtered by interventional studies. The full search strategy, including specific queries, is included in the online supplementary materials.^19^ Searches in the VAERS repository of AE data were not performed as these data are self-reported and not systematically collected.

### Identification of full publications of CHIS

We used two strategies to identify (1) publications listed in the ClinicalTrials.gov record for each included CHIS and (2) additional articles that were not listed in the ClinicalTrials.gov record, with a PubMed search using each National Clinical Trial (NCT) number. Articles were included if they were peer-reviewed and reported AEs in the CHIS linked by NCT number. Articles published before the date the record was first posted on ClinicalTrials.gov were excluded, as were articles reporting secondary analyses of data from registered CHIS. Conference abstracts, unpublished reports and other forms of grey literature were not evaluated.

### Study categorisation

Studies were categorised according to recruitment status to differentiate between those that were still recruiting, ongoing, suspended and/or terminated, withdrawn, completed or of unknown status (missing updates). Studies that had been completed were further grouped by whether they posted results on ClinicalTrials.gov and whether a corresponding published article discussing the results of the study was listed within the ClinicalTrials.gov study record or identifiable through a PubMed search for the record’s NCT number.

### Screening and data extraction process

Each record was screened independently by two of six investigators. Titles and study descriptions were reviewed to evaluate whether the record described an CHIS. Where feasible, data were automatically extracted from the AACT database. The query used to extract data from the AACT database is available in online supplemental file 2. Data that were extracted automatically were independently reviewed by at least two reviewers for verification. For each trial record identified by the search query, the number of volunteers challenged, infected, with AEs and with SAEs were calculated via additional queries to the database that provided these sums. Data that could not be extracted automatically were extracted manually by two reviewers working independently. For records with a corresponding published article discussing results from the same study, data were likewise extracted manually. In instances where multiple publications were associated with an NCT ID, data were extracted manually from all of them. If the same number was reported differently in different publications, data were recorded as a range to account for this variability in reporting. Any discrepancies in screening or data extraction were either resolved by discussion among the reviewers or by JA-P or DT. Participant flow and AE data were not extracted from associated publications if results were not posted on the ClinicalTrials.gov record, as data from both a ClinicalTrials.gov record and a publication are necessary for comparison. Studies registered on ClinicalTrials.gov less than 1 year before 15 April 2023 (the date of our last query of the database) were excluded.

### Assessment of risk of bias in the selection of the reported result

The risk of bias in the selection of the reported result was assessed using domain 5 of the Cochrane Risk of Bias 2.0 tool.^20^ Assessments were performed by DT and confirmed by one of six reviewers. Disputes were resolved by JA-P or DT.

### Synthesis methods

ClinicalTrials.gov records that posted results and included at least one publication were included in data synthesis. Data were tabulated to create summary statistics by relevant parameters. We calculated the prevalence of discrepancies between reporting in ClinicalTrials.gov records and associated publications. Data were analysed by study sponsor type (private, public and public-private partnership), cohort size (first, second, third and fourth quartiles) and risk of bias in the selection of the reported result (low, some concerns and high). Where applicable, data for the number of volunteers challenged, infected, with AEs or with SAEs were recorded as a range to account for ambiguous reporting.

Studies sponsored by government organisations were categorised as public, studies sponsored by private for-profit or not-for-profit organisations were categorised as private and studies sponsored by an organisation that was formed as an independent collaborative partnership between a governmental organisation and a private organisation were categorised as public-private partnerships.

### Statistical analyses

Discrepancies were defined as any instance in which the same metric (number challenged, infected, with AEs or with SAEs) was unambiguously reported differently in the record and its associated publications. Records with data recorded as a range due to ambiguous reporting or data that were omitted in one source but not another were excluded from statistical analyses. ORs and 95% CIs were used post hoc to compare the likelihood of discrepancy. This method was chosen as it allows a comparison of the frequency of discrepancy between the trial record and its associated publications. The relationship between study size, risk of bias and sponsor type was evaluated post hoc using one-way ANOVAs for continuous data and χ^2^ tests of independence for categorical data. These methods were chosen as they allow comparison of the number of records in each subgroup of analysis, and a significant result would alert us to an imbalanced frequency for at least one subgroup. Statistical significance was defined at a 5% level for all analyses.

## Results

### Study selection

**Figure 1:**
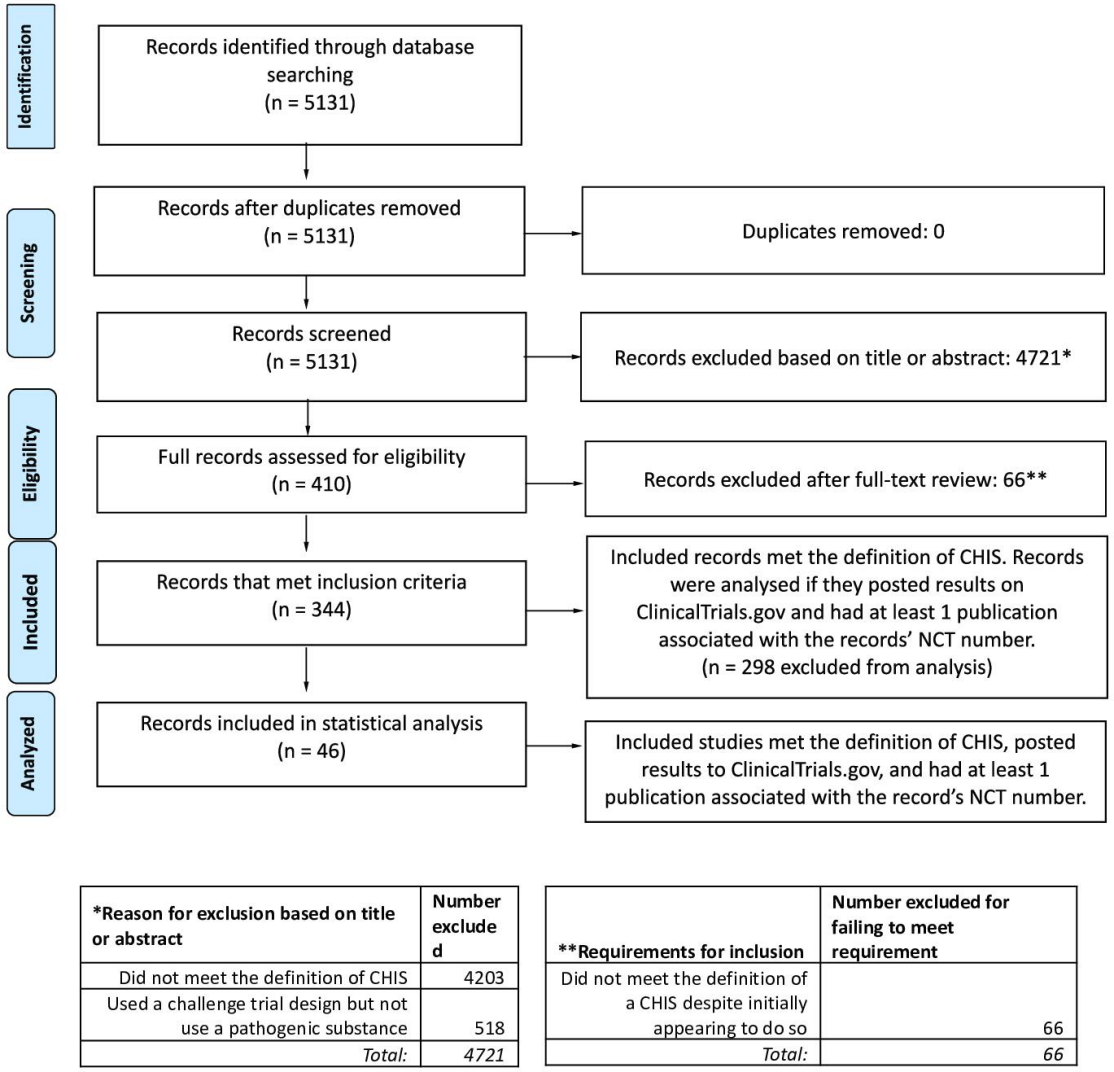
Preferred Reporting Items for Systematic Reviews and Meta-Analyses flow chart. AACT API, Aggregated Analysis of ClinicalTrials.gov application programming interface; CHIS, controlled human infection studies; NCT, National Clinical Trial.

The search returned 5131 records, of which 4721 records were excluded during screening (figure 1). The remaining 410 records were screened in full, of which 66 were excluded for not meeting the definition of an CHIS. The remaining 344 records met the inclusion criteria. A complete list of records excluded for these reasons is included in the online supplementary materials.

### Reporting in individual CHIS

Among the 344 included CHIS, 264 (76.7%) were completed, 13 (3.8%) were active and not recruiting, 22 (6.4%) were either recruiting or enrolling by invitation, 12 (3.5%) were terminated and 16 (4.7%) were of unknown status. Among all 264 completed CHIS, 66 (25.0%) posted results and 156 (59.1%) listed at least one published article. Among the 344 included CHIS, there were 46 studies (13.4%) that both posted results and linked to a publication and 209 studies (60.8%) that had results available in some form (either as results posted on ClinicalTrials.gov or by linking to a publication) (online supplemental table 5).

### Challenges, infections and AE reporting

Among completed CHIS, 46 ClinicalTrials.gov records both reported results and listed at least one associated publication, with 195 associated publications listed in total (median 1, range 1–82 publications per CHIS). The most common challenge agents were *Plasmodium* species (17 records), influenza (8 records), respiratory syncytial virus (4 records) and rhinovirus (4 records). Data were not extracted from the 474 associated publications whose ClinicalTrials.gov record did not post results. Where applicable, data are provided as ranges to account for ambiguous reporting. A total of 3574 volunteers were enrolled, of whom between 2998 (83.9%) and 3131 (87.6%) were challenged with a pathogen, and between 1297 (41.4–43.3%) and 1608 (51.4–53.6 %) were reported to have laboratory-confirmed infection or symptoms diagnostic for infection. In associated publications, the total reported enrolment was 3399, with 2615 (76.9%) to 2620 (77.1%) challenged volunteers and 1437 (54.8%–54.9%) to 1455 (55.5%–55.6%) confirmed infections. ClinicalTrials.gov records reported that 1921 (61.4%–64.1%) to 2166 (69.2%–72.2%) volunteers experienced at least one AE, whereas associated publications reported 1532 (58.5%–58.6%) to 1730 (66.0%–66.2%) volunteers experienced at least one AEs. Of note, 29 SAEs were reported in ClinicalTrials.gov records and 25 SAEs were reported in associated publications (online supplemental table 1a, online supplemental table 1b). The percentage of volunteers with AEs appeared to increase over time, though the trend was inconsistent (online supplemental figure 1).

### Comparison of results reported in ClinicalTrials.gov records versus associated publications

Completed records that both posted results on ClinicalTrials.gov and linked to a published article discussing results were compared with records that identified discrepancies in data reporting. Among the included records, 23 were sponsored by private organisations, 18 by public organisations and 5 by public-private partnerships, with 60, 127 and 8 associated publications, respectively (online supplemental table 2a, online supplemental table 2b). Results were posted for 25.7%, 14.6% and 50.0% of privately sponsored records, publicly sponsored records and public-private partnerships, respectively. Records were divided into quartiles by the number of volunteers enrolled, with the first, second, third and fourth quartiles being 6–26 volunteers, 27–58 volunteers, 59–79 volunteers and 80–440 volunteers, respectively (online supplemental table 3a). Of note, 26 ClinicalTrial.gov records had low risk, 1 had some concern and 19 had a high risk of bias related to the selection of the reported result (online supplemental table 4a).

#### Relationships between variables

To evaluate the relationship between sponsor type, risk of bias in selection of the reported result and study cohort size, several post-hoc analyses were performed. The relationship between sponsor type and study size was evaluated by a one-way ANOVA, which did not indicate a significant relationship (F=2.62, df=2, p=0.08). The relationship between the risk of bias in the selection of the reported result and study size was evaluated by a one-way ANOVA, which did not indicate a significant relationship (F=0.20, df=2, p=0.82). The relationship between sponsor type and risk of bias in the selection of the reported result was evaluated by a χ^2^ test for independence, which did not indicate a significant relationship (X=0.69, df=8, p=0.99).

#### Volunteers challenged

Twenty-eight records were included in statistical analysis for discrepancies among the number of volunteers challenged, with discrepancies observed in 14.3%. Publicly sponsored studies were more likely than other studies to have discrepancies (OR: 2.43, 95% CI: 1.10 to 5.37). Studies in the second quartile (OR: 3.00, 95% CI: 1.35 to 6.64) and third quartile (OR: 5.00, 95% CI: 2.25 to 11.11) of study size were more likely than studies in the other quartiles to have discrepancies. Studies with a low risk of bias in the selection of the reported result were more likely than studies with some concerns or studies with a high risk of bias to have discrepancies (OR: 3.00, 95% CI: 1.24 to 7.25), and studies with a high risk of bias were less likely than studies with some concerns or a low risk of bias to have discrepancies (OR: 0.39, 95% CI: 0.17 to 0.94) (table 1). Among the four records with discrepancies in the number of volunteers challenged, ClinicalTrials.gov records were more complete in all instances.

**Table 1:** OR comparisons: prevalence of discrepancy between ClinicalTrials.gov records and associated publications among the reported number of volunteers

	Challenged			Infected			With AEs			With SAEs		
*Sponsor type*	OR	95% CI LB	95% CI UB	OR	95% CI LB	95% CI UB	OR	95% CI LB	95% CI UB	OR	95% CI LB	95% CI UB
Private vs not private	0.85	0.38	1.87	1.45	0.64	3.33	0.80	0.46	1.40	0.41	0.15	1.17
Public vs not public	2.43*	1.10*	5.37*	1.40	0.63	3.09	1.75	0.99	3.10	4.89*	1.65*	14.50*
Public-private vs not public-private	0.00	N/A	N/A	0.00	N/A	N/A	0.70	0.40	1.22	0.00	N/A	N/A
*Study size*												
Fourth quartile vs not fourth	0.00	N/A	N/A	0.00	N/A	N/A	0.30*	0.17*	0.54*	0.00	N/A	N/A
Third quartile vs not third	5.00*	2.25*	11.11*	10.67*	4.23*	26.88*	N/A**	N/A	N/A	1.44	0.55	3.73
Second quartile vs not second	3.00*	1.35*	6.64*	0.00	N/A	N/A	0.55*	0.31*	0.96*	2.08	0.82	5.31
First quartile vs not first	0.00	N/A	N/A	1.40	0.63	3.09	0.18*	0.10*	0.33*	1.44	0.55	3.73
*Risk of bias*												
Low vs not low	3.00*	1.24*	7.26*	0.56	0.24	1.29	0.80	0.46	1.40	0.36	0.13	1.03
Some concern vs not some concern	0.00	N/A	N/A	N/A†	N/A	N/A	0.00	N/A	N/A	0.00	N/A	N/A
High vs not high	0.39*	0.17*	0.94*	0.45	0.19	1.05	2.00*	1.13*	3.54*	3.17*	1.10*	9.14*

Higher OR indicates a higher prevalence of discrepancy in the reference group.

* Significant at an alpha level of 0.05.

† The prevalence of discrepancy in the reference groups in these comparisons was 100%, precluding OR analysis.

#### Volunteers infected

Twenty-two records were included in statistical analysis for discrepancies among the number of volunteers with signs of infection after the challenge, with discrepancies observed in 13.6%. Studies in the third quartile of study size were more likely than studies in the other quartiles to have discrepancies in the number of volunteers infected after the challenge (OR: 10.67, 95% CI: 4.23 to 26.88). All records with some concerns for bias had discrepancies in the number of volunteers infected after the challenge (table 1). Of the three records with discrepancies in the number of volunteers infected, associated publications were more complete than ClinicalTrials.gov in all instances.

#### AE reporting

Twenty-one records were included in statistical analysis for discrepancies among the number of volunteers with AEs, with discrepancies observed in 61.9%. All 11 studies in the third quartile of study size had discrepancies in the number of volunteers with AEs. Studies with a high risk of bias in selection of the reported result were more likely than studies with a low risk of bias or some concerns to have discrepancies in the number of volunteers with AEs (OR: 2.00, 95% CI: 1.13 to 3.54). Studies in the first quartile (OR: 0.18, 95% CI: 0.10 to 0.33), second quartile (OR: 0.55, 95% CI 0.31 to 0.96) and fourth quartile (OR: 0.30, 95% CI: 0.17 to 0.54) of study size were less likely than studies in the third quartile to have discrepancies in the number of volunteers with AEs (table 1). Of the 13 records with discrepancies in the number of volunteers with AEs, ClinicalTrials.gov records were more complete in 9 (69.2%) instances and associated publications were more complete in 4 (30.8%) instances.

#### SAE reporting

Thirty-four records were included in statistical analysis for discrepancies among the number of volunteers with SAEs, with discrepancies observed in 8.8%. Studies sponsored by publicly funded organisations were more likely than other trials to have discrepancies in the number of reported SAEs (OR: 4.89, 95% CI: 1.65 to 14.50). Studies with a high risk of bias were more likely than studies with a low risk of bias or some concerns to have discrepancies (OR: 3.17, 95% CI: 1.10 to 9.14) (table 1). Of the three records with discrepancies in the number of volunteers with SAEs, ClinicalTrials.gov records were more complete in two (66.7%) instances and associated publications were more complete in one (33.3%) instance.

#### Overall completeness of AE reporting

Across 71 studies (66 completed and 5 terminated) that reported results on ClinicalTrials.gov and in associated publications, between 696 and 818 participants experienced AEs that were reported in ClinicalTrials.gov records but not in associated publications. Between 257 and 348 participants experienced AEs that were reported in associated publications but not in ClinicalTrials.gov records. The median time to post results in the ClinicalTrial.gov record was 1382 days (IQR 774–2191). Notably, 94% of ClinicalTrials.gov records reported AEs individually, rather than grouping related symptoms into sets, compared with 60.9% of associated publications. There were 6–13 AEs that were reported after rechallenge in ClinicalTrials.gov records that were not reported in associated publications.

There were five participants who each experienced one SAE that was reported in ClinicalTrials.gov records but not in any associated publication: fractured wrist, breast cancer in situ, peripheral parasitemia, ruptured Achilles tendon and pulmonary embolism. One record (NCT01024686) was terminated and so is not included in analyses, but is included in this summary for completeness. The relatedness of these SAEs to challenge was not discussed in their respective records. No participants experienced SAEs that were reported in associated publications but not in ClinicalTrials.gov records (online supplemental table 6). An additional six participants experienced SAEs that were reported in ClinicalTrials.gov records that had no associated publications: asthma, rhabdomyolysis, acute coronary syndrome, acute psychosis and foetal death.

## Discussion

In this analysis, we compared results available in ClinicalTrials.gov records with results available in these records’ associated publications and quantified the prevalence of discrepancies between these sources. The likelihoods of discrepancies among the number of volunteers challenged, infected, with AEs and with SAEs were 14.3%, 13.6%, 61.9% and 8.8%, respectively. Among all records eligible for analysis, 39.1% had some discrepancy. We also identified five SAEs that were reported in ClinicalTrials.gov records and not discussed in any publication of the results.

Publicly funded trials and trials with a high risk of bias related to the selection of the reported result were most likely to have discrepancies in reported SAEs. Trials in the third quartile of study size and trials with a high risk of bias related to the selection of the reported result were more likely to have discrepancies in reported AEs than studies in other study size quartiles and other risks of bias, respectively. There were no significant relationships identified between sponsor type, risk of bias and study size in the occurrence of discrepancies. Data on the amount of funding trials received were not available, and it is possible that this would be a relevant factor. For example, if large, privately funded trials typically have more resources than smaller, publicly funded trials, they may have the budget to ensure proper reporting, which may have explained these results. The lower prevalence of discrepancies observed in smaller studies may reflect the relative ease of consistent reporting when the overall number of events to report is low. It is also possible that ClinicalTrials.gov reporting is more complete due to constraints on the sharing of data in the process of preparing manuscripts for peer review and publication. At the same time, 59% of trials completed listed at least one publication in their record while only 25% had posted results despite their mandate to do so,^21^ which may contextualise the relative ease of publishing a limited amount of relevant data in a peer-reviewed journal as opposed to a complete set of results in a clinical trial record.

Issues with data reporting in clinical trials are not isolated to CHIS, with a body of literature showing that reporting discrepancies are prevalent in other types of clinical trials. A recent review of trials registered on ClinicalTrials.gov in Canada found that 32% neither reported their results nor discussed them in publications.^22^ A review of outcome-related discrepancies between registry entries and published reports in orthodontic RCTs identified discrepancies in 47% of publications,^23^ while a review of oncology trials identified a 63% discrepancy in secondary outcomes described in protocols compared with final results.^24^ A random sample of 300 trials posted on ClinicalTrials.gov identified a discrepancy prevalence of 32% for SAEs in records compared with SAEs in publications of results.^25^ A study investigating reporting discrepancies in a random sample of 110 phase 3 or 4 trials with results posted on ClinicalTrials.gov found that 20% of trials have inconsistencies in reported primary outcomes.^26^ Our findings regarding discrepancies in the reporting of CHIS are therefore similar to those observed in other types of clinical research.

We previously reported that AE reporting in CHIS is inconsistent.^10^ Though this is not unique to CHIS, there is arguably a greater benefit to consistent reporting due to the increased value of data aggregation in a field of research where studies are typically small and where key benefits include providing results more quickly and with fewer volunteers than alternative trial designs (eg, vaccine field trials). In particular, data on the number of volunteers who are challenged, become infected, and subsequently experienced AEs and/or SAEs must be reported clearly, especially if related to challenge or other study procedures. Consistent reporting is important for a wide range of clinical studies. The European Union’s Clinical Trials Information System (EU CTIS) clinical trial registry provides an example of a platform that facilitates consistent reporting by integrating trial registration with outcome reporting. CTIS registration requires expected AEs to be predefined. Clear reporting of which expected and unexpected AEs are noted for volunteers who do and do not receive a challenge organism would help to ensure accurate descriptions of what volunteers experience.

A key finding of the current study is that reporting is clearer in a database than in publications because it is more comprehensive and easier to compare due to standardisation. Repositories also facilitate finding and aggregating data for future studies. Our experience aggregating data for the current study highlights the ClinicalTrials.gov AACT API as a powerful tool that promotes efficient data sharing with minimal modifications. In the online supplemental methods, we provide an open-source programme that was used to generate our dataset and can be used by others.^27^ Our focus on ClinicalTrials.gov is not to imply that it should be the repository for all clinical trials but rather to highlight aspects of its reporting requirements. On the data entry side, it is relatively simple to add fields by arm for AE data. On the data reuse side, the AACT API is powerful and easy to use, with a well-documented schema to simplify finding a data field of interest. These functionalities of ClinicalTrials.gov provide a simple method for sharing AE data and could be used more widely. It serves as a strong example of a platform that fulfils FAIR data-sharing principles. We recommend that CHIS researchers add data fields alongside the AE and SAE outcomes for the number of volunteers challenged and infected in each arm. We make these recommendations with the goal of (a) ensuring CHIS fulfil FAIR data-sharing practices to the fullest degree and (b) maximising the contribution each CHIS volunteer makes to science by facilitating the ease with which other researchers may access and find new insights from their data.

Our study has the following limitations: (1) not every relevant record may have been caught by the ClinicalTrials.gov search query used; (2) not every publication associated with each record may have been identified by NCT number; (3) our results may have been influenced by publication bias, which favours significant results; (4) our data likely exhibit heterogeneity arising from aggregation across different infectious agents and (5) only ClinicalTrials.gov was evaluated, and it is possible that trials that would have met inclusion criteria are registered in other databases.

In conclusion, we found that around 40% of CHIS had at least one discrepancy between results reported in ClinicalTrials.gov records and publications describing the same results. Rather than this being unique to CHIS, we note that similar levels of suboptimal reporting have been observed in other types of trials. As a general trend, publicly funded trials in the middle quartiles of cohort size were more likely to have discrepancies in reporting, which may reflect the resources typically available to large, privately funded trials or the relative ease of reporting in smaller trials. To address these issues, we propose minimal amendments to CHIS researchers’ data entry workflow in ClinicalTrials.gov to facilitate automatic aggregation of CHIS data via the AACT API and highlight the EU CTIS as an example of an integrated registration system that facilitates clear and consistent reporting. The data produced by CHIS are unique and valuable. However, when not reported clearly, the value of these data becomes confined to the specific study in which they were collected. This not only limits their broader utility but also does a disservice to CHIS volunteers and the public they serve through their participation in clinical research when data are siloed within different research groups and filed away at the end of a study. Our results show that by making data available in a repository like ClinicalTrials.gov, study investigators allow others to reuse data in new and innovative ways that are not easily performed when data are only available in publications, which maximises the public good volunteers can do through their participation. For these reasons, it is arguably imperative that volunteer outcomes be accurately described in publicly available repositories so that study data can be used as broadly and effectively as possible.

## supplementary material

10.1136/bmjopen-2024-085250 online supplemental file 1

10.1136/bmjopen-2024-085250 online supplemental file 2

10.1136/bmjopen-2024-085250 online supplemental file 3

10.1136/bmjopen-2024-085250 online supplemental file 4

## Data Availability

Data are available in a public, open access repository.
