# Supplementary material for: An analysis of controlled human infection studies registered on ClinicalTrials.gov
Source: BMJ Open. 2025 Feb 7;15(2):e085250. doi: 10.1136/bmjopen-2024-085250 (PMC11808890; doi:10.1136/bmjopen-2024-085250)
Supplement: online supplemental file 1 [file bmjopen-15-2-s001.docx]

**ClinicalTrials.gov Systematic Review Protocol**

*Objectives*

The proposed review aims to investigate the use of ClinicalTrials.gov for CHIS registration and data reporting, and how this compares with data reporting in corresponding published articles describing CHIS. With the goal of evaluating the current use of ClinicalTrials.gov as a registration platform and recommending guidelines for future use, the present review seeks to answer the following research questions:

1. How many CHIS registered on ClinicalTrials.gov have at least one published article to report their results and how many post results to the CT record?
   1. Reported in Section 3.2
2. Of those that post results, how many volunteers were challenged and infected? How many experienced AEs and SAEs?
   1. Reported in Section 3.3
3. How do these results compare with reporting in published articles, specifically:
   1. Are there discrepancies in the number of reported volunteers challenged?
      1. Reported in Section 3.4 B
   2. Are there discrepancies in the number of reported volunteers positive for infection?
      1. Reported in Section 3.4 C
   3. Are there discrepancies in AE reporting?
      1. Reported in Section 3.4 D
   4. Are there discrepancies in SAE reporting?
      1. Reported in Section 3.4 E
   5. Are there discrepancies in the level of detail of reported adverse events?
      1. Reported in Section 3.4 F
4. Based on the quality of data reporting and record metadata (such as length of trials and number of trials that are not completed), do CHIS make effective use of ClinicalTrials.gov?
   1. Reported in Section 3.4 F

*Search Strategy*

A query was performed on 4/15/23 using the AACT API with the following terms:

select * from studies where

study_type = 'Interventional'

and

enrollment < 1000

and

study_first_submitted_date < '2022-06-30'

and

(

(official_title ilike '%challenge%') or

(official_title ilike '%immunization%' and official_title ilike '%sporozoites%') or

(official_title ilike '%human%' and official_title ilike '%carriage%') or

(official_title ilike '%infection%' and

(official_title ilike '%controlled%' or official_title ilike '%experimental%' or official_title ilike '%induced%')) or

(official_title ilike '%efficacy%' and official_title ilike '%vaccine%') or

(official_title ilike '%human%' and official_title ilike '%exposure%') or

(official_title ilike '%healthy%' and

(official_title ilike '%naïve%' or official_title ilike '%naive%')) or

(official_title ilike '%competitive%' and official_title ilike '%carriage%')

OR

(brief_title ilike '%challenge%') or

(brief_title ilike '%immunization%' and brief_title ilike '%sporozoites%') or

(brief_title ilike '%human%' and brief_title ilike '%carriage%') or

(brief_title ilike '%infection%' and

(brief_title ilike '%controlled%' or brief_title ilike '%experimental%' or brief_title ilike '%induced%')) or

(brief_title ilike '%efficacy%' and brief_title ilike '%vaccine%') or

(brief_title ilike '%human%' and brief_title ilike '%exposure%') or

(brief_title ilike '%healthy%' and

(brief_title ilike '%naïve%' or brief_title ilike '%naive%')) or

(brief_title ilike '%competitive%' and brief_title ilike '%carriage%')

OR

(acronym ilike '%challenge%') or

(acronym ilike '%human%')

OR

nct_id IN

(select s.nct_id from studies s, keywords k where

s.nct_id = k.nct_id and k.name ilike '%challenge%')

OR

nct_id IN

(select s.nct_id from studies s, detailed_descriptions d where

s.nct_id = d.nct_id and

((d.description ilike '%challenge%') and

(d.description ilike '%infection%' or

d.description ilike '%controlled%' or

d.description ilike '%experimental%')))

OR

nct_id IN

(select s.nct_id from studies s, brief_summaries b where

s.nct_id = b.nct_id and

((b.description ilike '%challenge%') and

(b.description ilike '%infection%' or

b.description ilike '%controlled%' or

b.description ilike '%experimental%')))

)

For record identified by this query, an additional query was performed to aggregate AE data:

select cv.nct_id, cv.number_of_nsae_subjects, cv.minimum_age_num, cv.maximum_age_num,

dg.design_groups,

iv.interventions,

oap.p_value,oac.ci_percent,

srp.pmid,src.citation,

pf.recruitment_details,

rd.AE_Count,rd.SAE_Count,rd.Mortality_Count,

re.Num_AEs_described

from (

select calculated_values.nct_id, calculated_values.number_of_nsae_subjects, calculated_values.minimum_age_num, calculated_values.maximum_age_num

from calculated_values

where calculated_values.nct_id = '{}' ) as cv

left join (

select design_groups.nct_id, string_agg(design_groups.description,'; ') as design_groups

from design_groups

group by design_groups.nct_id) as dg

on cv.nct_id = dg.nct_id

left join (

select interventions.nct_id, string_agg(interventions.description,'; ') as interventions

from interventions

group by interventions.nct_id) as iv

on cv.nct_id = iv.nct_id

left join (

select outcome_analyses.nct_id, string_agg(CAST(outcome_analyses.p_value as VarChar),'; ') as p_value

from outcome_analyses

group by outcome_analyses.nct_id) as oap

on cv.nct_id = oap.nct_id

left join (

select outcome_analyses.nct_id, string_agg(CAST(outcome_analyses.ci_percent as VarChar),'; ') as ci_percent

from outcome_analyses

group by outcome_analyses.nct_id) as oac

on cv.nct_id = oac.nct_id

left join (

select study_references.nct_id, string_agg(CAST(study_references.pmid as VarChar),'; ') as pmid

from study_references

group by study_references.nct_id) as srp

on cv.nct_id = srp.nct_id

left join (

select study_references.nct_id, string_agg(CAST(study_references.citation as VarChar),'; ') as citation

from study_references

group by study_references.nct_id) as src

on cv.nct_id = src.nct_id

left join (

select participant_flows.nct_id, string_agg(CAST(participant_flows.recruitment_details as VarChar),'; ') as recruitment_details

from participant_flows

group by participant_flows.nct_id) as pf

on cv.nct_id = pf.nct_id

left join (

select reported_events.nct_id, COUNT(DISTINCT reported_events.adverse_event_term) AS Num_AEs_described

from reported_events

group by reported_events.nct_id) as re

on cv.nct_id = re.nct_id

left join(

select reported_event_totals.nct_id,

sum(case when reported_event_totals.classification = 'Total, other adverse events' then

reported_event_totals.subjects_affected else 0 end) as AE_Count,

sum(case when reported_event_totals.classification = 'Total, serious adverse events' then

reported_event_totals.subjects_affected else 0 end) as SAE_Count,

sum(case when reported_event_totals.classification = 'Total, all-cause mortality' then

reported_event_totals.subjects_affected else 0 end) as Mortality_Count

from reported_event_totals

group by reported_event_totals.nct_id) as rd

on cv.nct_id = rd.nct_id

This search returned 5,131 results. A filter by study size was applied using a cohort size of 1,000 as an upper limit. This is based on our previous systematic review, in which the largest CHIS identified had a cohort size of 437. This number was rounded to 500 and doubled to 1000 to ensure a conservative ceiling on sample size to maximize the number of eligible CHIS. Interventional studies were used as filter criteria because CHIS are rarely classified as another type of study.

*Systematic review protocol*

Titles and brief descriptions for 5,131 results (ClinicalTrials.gov records) were screened for inclusion by 2 of 6 reviewers. In instances in which the title or brief description did not provide sufficient evidence for challenge, the arms of the trials were checked for challenge phases. Records that described a study that intentionally exposed human participants to an infectious pathogen for the purpose of modeling a named human disease were included. Records that did not intentionally expose a human cohort to an infectious pathogen (such as studies involving a live vaccine that was not given with intent to challenge, studies using non-infectious challenge agents, and studies that performed noxious challenges with lipopolysaccharide or endotoxin) were excluded.

*Eligibility criteria*

Clinical trial records will be reviewed for the following criteria:

1. Trial intentionally exposes at least one volunteer to an infectious pathogen

Clinical trial records will be excluded for the following criteria:

1. Trial record is not in english
2. Trial record does not have sufficient information to determine the procedure and primary outcomes of the trial
3. Trial does not intentionally expose at least one volunteer to an infectious pathogen.

Trials that expose volunteers to live-attenuated pathogens will be included, unless the agent used is explicitly attenuated to the degree of being entirely non-pathogenic. Non-pathogenic agents often used in challenge studies, such as lipopolysaccharide or gluten, will not be included. In the current definition, an infectious agent must be a biological agent with the capacity to reproduce and cause infection in its wild-type, unattenuated form.

*Methods for identifying full-text articles*

Because ClinicalTrials.gov lists publications of trial results as part of the record, full-text articles will be identified from the list given in the record. All publications listed in the ClinicalTrials.gov record will be evaluated. A PubMed search for the trial’s NCT number will be performed to identify publications not listed in the ClinicalTrials.gov record.

*Statistical analysis plan*

Trials that have been completed or are currently ongoing will be included in data analysis. Trials that have not yet begun, have been withdrawn, or were completed within 1 year of the date data collection was completed (4/15/23) will be included but not analyzed.

The unit of analysis will be the rate of discrepancy between reporting in ClinicalTrials.gov records and associated publications. Data will be divided into groups by sponsor type (private, public, and public-private partnership), study size (1st, 2nd, 3rd, and 4th quartiles), and risk of bias (low, some concerns, high).

Odds ratios and 95% confidence intervals will be used to evaluate significance between the rates of discrepancy with each subgroup compared to the remainder of its group. These analyzes will be performed post-hoc and were not pre-registered.

*Data items:*

Reported AE’s will be assessed for the following qualities:

a) reporting of frequency of AE assessment,

b) duration of follow up for AEs,

c) investigators attribution of the cause of SAEs, categorized as related or unrelated to challenge,

d) what proportion of AEs determined by the investigators to be unexpected

The following data will be automatically extracted from CT records:

- Status of trial (Active but not recruiting, Completed, Enrolling by invitation, Not yet recruiting, Terminated, Unknown status, or Withdrawn)
- The date the study was posted
- The date results were posted posted
- Study type
- Enrollment
- Minimum age
- Maximum age
- Number of volunteers with at least 1 SAE
- Number of volunteers with at least 1 AE
- All cause mortality
- Number of potential AEs described
- Study sponsor

The following data will be manually extracted from CT records:

- Number of volunteers in challenge groups
- Number of volunteers in challenge groups that became infected with the pathogen
- Are AEs detailed individually? (true/false)
- Total number of publications
- Number of publications automatically indexed
- Number of volunteers with AEs prior to challenge
- Number of volunteers with AEs after challenge
- Number of volunteers with AEs after rechallenge

The following data will be manually extracted from associated publications:

- Study enrollment
- Number of volunteers in challenge groups
- Number of volunteers in challenge groups that became infected with the pathogen
- Number of volunteers with at least 1 SAE
- Number of volunteers with at least 1 AE
- All cause mortality
- Are AEs detailed individually? (true/false)
- Number of volunteers with AEs prior to challenge
- Number of volunteers with AEs after challenge
- Number of volunteers with AEs after rechallenge

Automatic data extraction will be reviewed by 2 of 6 reviewers. Manual data extraction for each included record and any publications associated with the record that discuss results of the study will also be performed by 2 of 6 reviewers. Conflicting data reported by different reviewers will be resolved by DT or JAP.

*Risk of bias in individual records:*

Risk of bias in individual records will be assessed using the Cochrane Risk of Bias 2.0 Tool by 2 of 6 reviewers. The Cochrane Risk of Bias 2.0 tool evaluates bais using separate algorithms to rate the risk of bias from 5 potential sources in publications. In the present study, bias arising from Domain 5, “Risk of bias in the selection of the reported result,” will be evaluated for ClinicalTrials.gov records and all associated publications. Results of these assessments for ClinicalTrials.gov records and associated publications will be combined for a single assessment given per ClinicalTrial.gov record. Disputes will be resolved by JAP or DT.

*Data synthesis:*

ClinicalTrials.gov records will be determined eligible for analysis if they post results and have an associated publication linked to their NCT number. Data will be tabulated to create summary statistics by relevant parameters as detailed in the analysis grouping below.

Analysis grouping:

- Trial recruitment status
  - Completed: The primary group used for analyzing data
  - Active, not recruiting: Used to analyze the number of current studies (and when they started, how long they’ve been in progress, estimated completion, etc.)
  - Recruiting (and Enrolling by invitation): Used to analyze the number of current studies (and when they started, how long they’ve been in progress, estimated completion, etc.)
  - Withdrawn (and Terminated): Used to analyze studies that were proposed (or even started) that were not finished, in order to identify and investigate any studies that exposed participants to a challenge agent, but did not finish the study or report results of the challenge
  - Unknown status: Studies that haven’t been updated within a certain timeframe; will be analyzed individually for available data
- Sponsor type
  - Private sponsorship: Private for-profit or not-for-profit organizations
  - Public sponsorship: Governmental organizations
  - Public-private partnership: An independent collaborative partnership between a governmental organization and a private organization
- Study size
  - 1st Quartile: The 1st quartile of enrollment size for all studies included in analysis
  - 2nd Quartile: The 2nd quartile of enrollment size for all studies included in analysis
  - 3rd Quartile: The 3rd quartile of enrollment size for all studies included in analysis
  - 4th Quartile: The 4th quartile of enrollment size for all studies included in analysis
- Risk of bias
  - Low risk of bias: A low risk of bias as determined by the algorithm provided Domain 5 of the Cochrane Risk of Bias 2.0 Tool, taking into account results reported in the ClinicalTrials.gov record and all publications associated with its NCT number.
  - Some concerns: Some concerns as determined by the algorithm provided Domain 5 of the Cochrane Risk of Bias 2.0 Tool, taking into account results reported in the ClinicalTrials.gov record and all publications associated with its NCT number.
  - High risk of bias: A high risk of bias as determined by the algorithm provided Domain 5 of the Cochrane Risk of Bias 2.0 Tool, taking into account results reported in the ClinicalTrials.gov record and all publications associated with its NCT number.
