# Supplementary material for: An analysis of controlled human infection studies registered on ClinicalTrials.gov
Source: BMJ Open. 2025 Feb 7;15(2):e085250. doi: 10.1136/bmjopen-2024-085250 (PMC11808890; doi:10.1136/bmjopen-2024-085250)
Supplement: online supplemental file 2 [file bmjopen-15-2-s002.docx]

select * from studies where

*This term selects all studies from the API, which are filtered by the following terms.*

study_type = 'INTERVENTIONAL'

*This term filters the search by only interventional studies, as CHIS would only be listed in ClinicalTrials.gov as interventional.*

and

enrollment < 1000

*This term filters the trials by an enrollment of 1,000 volunteers or fewer, which is a conservative filter as it is double the largest CHIS identified in our previous review.*

and

study_first_submitted_date < '2022-06-30'

and

*This term filters the search by studies posted before 6/30/2022 to try to make the search as replicable as possible, since ClinicalTrials.gov is a live database that is continually updated..*

(

(official_title ilike '%challenge%') or

(official_title ilike '%immunization%' and official_title ilike '%sporozoites%') or

(official_title ilike '%human%' and official_title ilike '%carriage%') or

(official_title ilike '%infection%' and

(official_title ilike '%controlled%' or official_title ilike '%experimental%' or official_title ilike '%induced%')) or

(official_title ilike '%efficacy%' and official_title ilike '%vaccine%') or

(official_title ilike '%human%' and official_title ilike '%exposure%') or

(official_title ilike '%healthy%' and

(official_title ilike '%naïve%' or official_title ilike '%naive%')) or

(official_title ilike '%competitive%' and official_title ilike '%carriage%')

*These terms search for the keywords given between each set of percentage signs (e.g.* '%challenge%'*) in official titles for ClinicalTrials.gov records.*

OR

(brief_title ilike '%challenge%') or

(brief_title ilike '%immunization%' and brief_title ilike '%sporozoites%') or

(brief_title ilike '%human%' and brief_title ilike '%carriage%') or

(brief_title ilike '%infection%' and

(brief_title ilike '%controlled%' or brief_title ilike '%experimental%' or brief_title ilike '%induced%')) or

(brief_title ilike '%efficacy%' and brief_title ilike '%vaccine%') or

(brief_title ilike '%human%' and brief_title ilike '%exposure%') or

(brief_title ilike '%healthy%' and

(brief_title ilike '%naïve%' or brief_title ilike '%naive%')) or

(brief_title ilike '%competitive%' and brief_title ilike '%carriage%')

*These terms search for the keywords given between each set of percentage signs (e.g.* '%challenge%'*) in brief titles for ClinicalTrials.gov records.*

OR

(acronym ilike '%challenge%') or

(acronym ilike '%human%')

*These terms search for the keywords given between each set of percentage signs (e.g.* '%challenge%'*) in acronyms for ClinicalTrials.gov records.*

OR

nct_id IN

(select s.nct_id from studies s, keywords k where

s.nct_id = k.nct_id and k.name ilike '%challenge%')

*These terms search for the keywords given between each set of percentage signs (e.g.* '%challenge%'*) in keywords for ClinicalTrials.gov records.*

OR

nct_id IN

(select s.nct_id from studies s, detailed_descriptions d where

s.nct_id = d.nct_id and

((d.description ilike '%challenge%') and

(d.description ilike '%infection%' or

d.description ilike '%controlled%' or

d.description ilike '%experimental%')))

*These terms search for the keywords given between each set of percentage signs (e.g.* '%challenge%'*) in descriptions for ClinicalTrials.gov records.*

OR

nct_id IN

(select s.nct_id from studies s, brief_summaries b where

s.nct_id = b.nct_id and

((b.description ilike '%challenge%') and

(b.description ilike '%infection%' or

b.description ilike '%controlled%' or

b.description ilike '%experimental%')))

)

*These terms search for the keywords given between each set of percentage signs (e.g.* '%challenge%'*) in brief summaries for ClinicalTrials.gov records.*
