# Supplementary figures and images for: An analysis of controlled human infection studies registered on ClinicalTrials.gov
Source: BMJ Open. 2025 Feb 7;15(2):e085250. doi: 10.1136/bmjopen-2024-085250 (PMC11808890; doi:10.1136/bmjopen-2024-085250)

# Percent of AEs over time

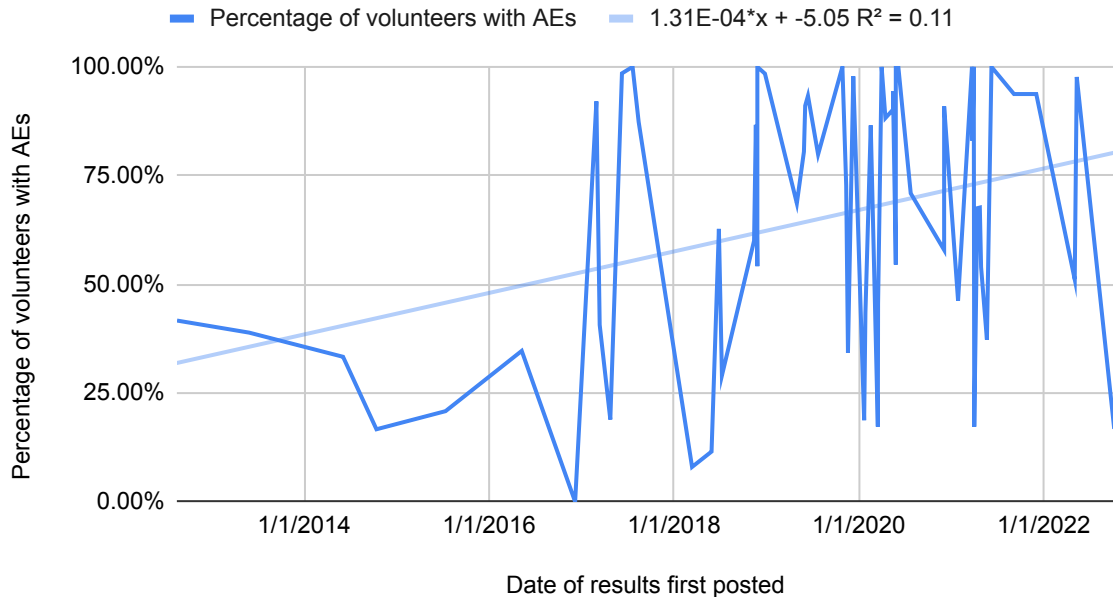

Supplement: online supplemental file 3 [file bmjopen-15-2-s003.pdf]
