# Supplementary material for: An analysis of controlled human infection studies registered on ClinicalTrials.gov
Source: BMJ Open. 2025 Feb 7;15(2):e085250. doi: 10.1136/bmjopen-2024-085250 (PMC11808890; doi:10.1136/bmjopen-2024-085250)
Supplement: Supplementary file 5 [file bmjopen-2024-085250.reviewer_comments.pdf]

# PEER REVIEW HISTORY

BMJ Open publishes all reviews undertaken for accepted manuscripts. Reviewers are asked to complete a checklist review form and are provided with free text boxes to elaborate on their assessment. These free text comments are reproduced below.

## ARTICLE DETAILS

### Title (Provisional)

An Analysis of Controlled Human Infection Studies Registered on ClinicalTrials.gov

### Authors

Toomey, Danny; Adams-Phipps, Jupiter; Wilkinson, James; Pietro , John; Littman, Jake; Kamenicek, Steffen; Kaufman, Daniel; Osowicki, Joshua; Roestenberg, Meta; Saldanha, Ian J; Jamrozik, Euzebiusz

---

## VERSION 1 - REVIEW

---

| | |
|---|---|
| **Reviewer** | **1** |
| **Name** | **Gresham, Gillian** |
| **Affiliation** | **Cedars-Sinai Medical Center Samuel Oschin Comprehensive Cancer Institute, Cancer Research Center for Health Equity** |
| **Date** | **23-Apr-2024** |
| **COI** | **No competing interests.** |

---

This was an interesting and well-written review summarizing the use of ClinicalTrials.gov or Controlled Human Infection Studies and comparing the reporting data in ClinicalTrials.gov records to the publications. While authors report a detailed and thorough analysis of the CHIS reporting data, the following suggestions may be helpful to

• Introduction:

o May be helpful to define what “small” is (range /average size) compared to other trials

o Line 23/24: Was this previous review also based on AE/SAE data as reported in clinicaltrials.gov? Or directly from reports?

o Again, is this within the definition within ClinicalTrials.gov? Would be helpful to know how these are classified earlier on.

o Would there be another source of AE reporting? VAERS?

o While authors provide a description of ClinicalTrials.gov and some of the features, further justification as to why it is important in this context and how it can be used as a tool to access AE data. Why ClinicalTrials.gov over other registries or other adverse event

databases? Would also want to know how CHIS are classified in ClinicalTrials.gov, whether they are considered “interventional” or listed as Applicable Clinical Trials (indicate whether AE reporting is required or optional for these studies), and if there are other databases that exist to access these data (e.g., would VAERS be another source?)

o Should briefly explain the consequences/ramifications of unclear and/or missing AE reporting in CHIS publications (following first sentence of last paragraph of introduction)

o More justification is needed to support objective 2- why is it important to compare participant flow? For both objectives, it is important to discuss the potential consequences of discrepancies, and overall rationale

• Methods

o Would be helpful to provide timeline in eligibility, since AE reporting was only available after 2008, unless authors clarify that studies also retrospectively entered AEs.

o Although the full search strategy is available in the supplementary material, some additional information of general terms/queries (e.g, interventional, used first submitted date <2022/6/30, and searched for words including “human, infection, controlled, etc.”

o No mention of whether conference abstracts or other publications were included for comparisons. Were any efforts made to search PubMED for related publications if not listed in the CT.gov record?

o What happens if there are multiple publications associated with one NCT number? How are these compared and is their a hierarchy (e.g., only compared with most recent? Article over abstract? Include all?) Were these all manually screened or extracted from the ClinicalTrial.gov publication tab, or from the AACT API? May help to expand on the methods here to help with reproducibility of these methods.

o May want to indicate earlier on in eligibility section that ClinicalTrials.gov records have to have available results to be included

o Last paragraph of methods: First time mentioning “number challenged”- may be worth defining and indicating how this would be identified in ClinicalTrials.gov for those who may not be familiar

o Would recommend elaborating on statistical methods (justify use of the different statistical tests/comparisons) or briefly summarize and indicate where to find additional details of statistical analysis in the protocol. (e.g., why use ANOVA models, and why not adjust for other potential variables in regression models? Could % agreement in results also be calculated or other methods for comparing discrepancies between sources? If a particular variable was missing in one source compared to the other, was this categorized as discrepant or not included in the analysis? )

May also be helpful to include a separate subtitle for the statistical analysis, to make easier to locate in the methods section.

Results/Discussions

• Study selection and flow diagram- please specify source of search/records (ClinicalTrials.gov via AACT) to avoid any confusion (e.g. records identified through database searching).

• Again, it is difficult to know the significance of the results, specifically for AE and SAE reporting, without knowing whether AE/SAE reporting are mandatory or required in ClinicalTrials.gov for CHIS. It may have been mentioned somewhere, but would recommend emphasizing in intro and then restating in results or discussion. This then leads to the question of whether it is recommended to be required for reporting.

• Would be helpful to provide a comparison of discrepancies in reporting for other trials based on other publications, to get a sense of range (e.g., how does the 13% discrepant AE data compare to other AE data?)

• Could the time of publication have affected the reporting of results, since AE/SAE reporting only became available in 2009

• Should define small vs. medium vs. large size trials, as this could be arbitrary. Only mention in conclusions (e.g., page 12, line 47). Also are the sizes for CHIS different than other trials considering the small sample sizes for these types of studies?

• Conclusion: While the value of ClinicalTrials.gov for accessing CHIS results and AE/SAE data are clear, it is difficult to know what the implications are to the public/patients and how this information can be used to improve future practice and health outcomes

Other comments: While abstract was generally complete and there may be space limitations, more detail in the methods and results would be helpful. The justification for the analysis could also be better aligned with the objectives of the study and results themselves (why is it important o evaluate use of ClinicalTrials.gov and why should the discrepancies between sources be assessed?)

The abstract could also align more closely with the manuscript body (intro/methods/results/conclusions).

---

## VERSION 1 - AUTHOR RESPONSE

Comments to the Author:
This was an interesting and well-written review summarizing the use of ClinicalTrials.gov or Controlled Human Infection Studies and comparing the reporting data in ClinicalTrials.gov records to the publications. While authors report a detailed and thorough analysis of the CHIS reporting data, the following suggestions may be helpful to

*Response: We thank the reviewer for their careful review and comments. Enclosed please find our point-by-point responses and clarifications.*

• Introduction:
o May be helpful to define what “small” is (range /average size) compared to other trials

*Response: We identified an average Controlled Human Infection Study (CHIS) size of 55 in our last review [Reference #10], compared to an average 1,262 volunteers for interventional studies listed on ClinicalTrials.gov [AACT API, Accessed 2024-06-26]. We have included this in lines 84-86.*

o Line 23/24: Was this previous review also based on AE/SAE data as reported in clinicaltrials.gov? Or directly from reports?

*Response: No, the previous systematic review was based solely on published data. This has been clarified in lines 88-89.*

o Again, is this within the definition within ClinicalTrials.gov? Would be helpful to know how these are classified earlier on.

*Response: The definition of SAE given at lines 89-91 is based on the Food and Drug Administration (FDA)'s definition and has been clarified accordingly.*

o Would there be another source of AE reporting? VAERS?

*Response: VAERS certainly has AE data, however these data include self reports by patients and are not systematically collected, and so we did not include this source. This has been clarified at lines 151-152.*

o While authors provide a description of ClinicalTrials.gov and some of the features, further justification as to why it is important in this context and how it can be used as a tool to access AE data. Why ClinicalTrials.gov over other registries or other adverse event databases? Would also want to know how CHIS are classified in ClinicalTrials.gov, whether they are considered “interventional” or listed as Applicable Clinical Trials (indicate whether AE reporting is required or optional for these studies), and if there are other databases that exist to access these data (e.g., would VAERS be another source?)

*Response: ClinicalTrials.gov was chosen as the data source because it is the largest registry of clinical trial data and its data are publicly accessible. We wanted to analyze systematically collected data, and because VAERS includes self-reported AE assessments by patients and is not gathered systematically, it was not included in our analysis. This has been clarified at lines 151-152. CHIS were not defined in ClinicalTrials.gov records. Records were reviewed and determined to be CHIS by the research team using the criteria given in lines 136-144. CHIS are listed as 'Interventional' in the ClinicalTrials.gov database.*

o Should briefly explain the consequences/ramifications of unclear and/or missing AE reporting in CHIS publications (following first sentence of last paragraph of introduction)

*Response: We have expanded on this point at lines 120-123.*

o More justification is needed to support objective 2- why is it important to compare participant flow? For both objectives, it is important to discuss the potential consequences of discrepancies, and overall rationale

*Response: We have expanded on this point at lines 127-129.*

• Methods

o Would be helpful to provide timeline in eligibility, since AE reporting was only available after 2008, unless authors clarify that studies also retrospectively entered AEs.

*Response: Our analysis did not differentiate between studies that retrospectively entered AEs after reporting became available after 2008 and studies first registered after 2008. If eligible studies registered prior to 2008 and retrospectively entered data after AE reporting became available, they were included in analysis.*

o Although the full search strategy is available in the supplementary material, some additional information of general terms/queries (e.g, interventional, used first submitted date <2022/6/30, and searched for words including "human, infection, controlled, etc."

*Response: We have added a sentence providing general terms in lines 149-150. In addition, we have included a document in the supplementary materials titled "AACT API Query Annotated" that annotates the terms used in AACT API query with rationales and the purposes of each term.*

o No mention of whether conference abstracts or other publications were included for comparisons. Were any efforts made to search PubMED for related publications if not listed in the CT.gov record?

*Response: Conference abstracts were not included, which has been clarified at line 160-161, and PubMed searches were performed for publications not listed in the ClinicalTrials.gov record as described in lines 155-157.*

o What happens if there are multiple publications associated with one NCT number? How are these compared and is their a hierarchy (e.g., only compared with most recent? Article over abstract? Include all?) Were these all manually screened or extracted from the ClinicalTrial.gov publication tab, or from the AACT API? May help to expand on the methods here to help with reproducibility of these methods.

*Response: We have expanded on this point at lines 178-181.*

o May want to indicate earlier on in eligibility section that ClinicalTrials.gov records have to have available results to be included

*Response: We have clarified this at lines 144-145.*

o Last paragraph of methods: First time mentioning "number challenged"- may be worth defining and indicating how this would be identified in ClinicalTrials.gov for those who may not be familiar

*Response: We have clarified this at lines 173-175.*

o Would recommend elaborating on statistical methods (justify use of the different statistical tests/comparisons) or briefly summarize and indicate where to find additional details of statistical analysis in the protocol. (e.g., why use ANOVA models, and why not adjust for other potential variables in regression models? Could % agreement in results also be calculated or other methods for comparing discrepancies between sources? If a particular variable was missing in one source compared to the other, was this categorized as discrepant or not included in the analysis? )
May also be helpful to include a separate subtitle for the statistical analysis, to make easier to locate in the methods section.

*Response: We have created a new subsection, 2.8, for statistical analyses and provided the suggested additional clarifications at lines 208-209, 211-212, and 214-216.*

Results/Discussions
• Study selection and flow diagram- please specify source of search/records (ClinicalTrials.gov via AACT) to avoid any confusion (e.g. records identified through database searching).

*Response: We have amended Figure 1 accordingly.*

• Again, it is difficult to know the significance of the results, specifically for AE and SAE reporting, without knowing whether AE/SAE reporting are mandatory or required in ClinicalTrials.gov for CHIS. It may have been mentioned somewhere, but would recommend emphasizing in intro and then restating in results or discussion. This then leads to the question of whether it is recommended to be required for reporting.

*Response: Yes, since January 2017, all NIH-funded trials are required to report results on ClinicalTrials.gov within 1 year after the primary completion date. This has been emphasized at lines 115-116 and 360-361.*

• Would be helpful to provide a comparison of discrepancies in reporting for other trials based on other publications, to get a sense of range (e.g., how does the 13% discrepant AE data compare to other AE data?)

*Response: These comparisons are provided at lines 367-372. The literature on reporting discrepancies is sparse, and so we have only identified one study who found that 32% of SAEs were reported differently in records compared to publications, which is substantially higher than the SAE discrepancy rate of 8.8% we observed.*

• Could the time of publication have affected the reporting of results, since AE/SAE reporting only became available in 2009

*Response: We have included a new supplemental Figure 1 that shows the percentage of volunteers with AEs as a function of the date results were first posted on ClinicalTrials.gov, with a regression fit of 1.31E-04*x + -5.05 and $R^2$ of 0.11. This shows that it is possible the*

*timing of when results were posted may have a slight impact on AE reporting, though results were inconsistent and do not support a definitive conclusion to this effect. This has been added at lines 248-249.*

• Should define small vs. medium vs. large size trials, as this could be arbitrary. Only mention in conclusions (e.g., page 12, line 47). Also are the sizes for CHIS different than other trials considering the small sample sizes for these types of studies?

*Response: We have clarified this at line 416. CHIS are usually smaller than other trials, though the sizes we refer to here is solely relative to CHIS.*

• Conclusion: While the value of ClinicalTrials.gov for accessing CHIS results and AE/SAE data are clear, it is difficult to know what the implications are to the public/patients and how this information can be used to improve future practice and health outcomes

*Response: We thank the reviewer for this question and have clarified this point at lines 422-429. Ultimately, our motivation in performing this study is to ensure complete reporting of data from CHIS volunteers. Given the inconsistent AE reporting in our last review, we aimed to understand how this problem could be addressed. Fundamentally, we hope to provide actionable solutions that ensure that data collected in CHIS are used as broadly as possible and not wasted through suboptimal reporting.*

Other comments: While abstract was generally complete and there may be space limitations, more detail in the methods and results would be helpful. The justification for the analysis could also be better aligned with the objectives of the study and results themselves (why is it important to evaluate use of ClinicalTrials.gov and why should the discrepancies between sources be assessed?)
The abstract could also align more closely with the manuscript body (intro/methods/results/conclusions).

*Response: We have amended this at lines 35-38 and 45-48.*

---

## VERSION 2 - REVIEW

---

| | |
|---|---|
| **Reviewer** | **1** |
| **Name** | **Gresham, Gillian** |
| **Affiliation** | **Cedars-Sinai Medical Center Samuel Oschin Comprehensive Cancer Institute, Cancer Research Center for Health Equity** |
| **Date** | **12-Dec-2024** |
| **COI** | |

---

The authors have adequately addressed reviewers' comments and their revisions have improved the strength of the manuscript. No further suggestions at this time.
